# Combining drought and submergence tolerance in rice: marker-assisted breeding and QTL combination effects

**DOI:** 10.1007/s11032-017-0737-2

**Published:** 2017-11-04

**Authors:** Shalabh Dixit, Anshuman Singh, Nitika Sandhu, Aditi Bhandari, Prashant Vikram, Arvind Kumar

**Affiliations:** 1 International Rice Research Institute (IRRI), DAPO Box 7777, Metro Manila, Philippines; 2 Rani Lakshmi Bai Central Agriculture University, Jhansi, India; 3 International Maize and Wheat Improvement Center (CIMMYT), Km. 45, Carretera México-Veracruz, El Batán, 56237 Texcoco, CP Mexico

**Keywords:** Rice, Drought, Yield, Submergence, QTL, MABB

## Abstract

TDK1 is a popular rice variety from the Lao PDR. Originally developed for irrigated conditions, this variety suffers a high decline in yield under drought conditions. Studies have identified three quantitative trait loci (QTLs) for grain yield under drought conditions, *qDTY*_*3.1*_, *qDTY*_*6.1*_, and *qDTY*_*6.2*_, that show a high effect in the background of this variety. We report here the pyramiding of these three QTLs with *SUB1* that provides 2–3 weeks of tolerance to complete submergence, with the aim to develop drought- and submergence-tolerant near-isogenic lines (NILs) of TDK1. We used a tandem approach that combined marker-assisted backcross breeding with phenotypic selection to develop NILs with high yield under drought stress and non-stress conditions and preferred grain quality. The effect of different QTL combinations on yield and yield-related traits under drought stress and non-stress conditions is also reported. Our results show *qDTY*_*3.1*_ to be the largest and most consistent QTL affecting yield under drought conditions, followed by *qDTY*_*6.1*_ and *qDTY*_*6.2*_, respectively. QTL class analysis also showed that lines with a combination of *qDTY*_*3.1*_ and *qDTY*_*6.1*_ consistently showed a higher tolerance to drought than those in which one of these QTLs was missing. In countries such as Lao PDR, where large areas under rice cultivation suffer vegetative-stage submergence and reproductive-stage drought, these lines could ensure yield stability. These lines can also serve as valuable genetic material to be used for further breeding of high-yielding, drought- and submergence-tolerant varieties in local breeding programs.

**Electronic supplementary material:**

The online version of this article (10.1007/s11032-017-0737-2) contains supplementary material, which is available to authorized users.

## Introduction

Drought and flooding are considered to be two of the most important abiotic stresses that affect rice production globally. A total of approximately 40 million hectares of rice area are affected by different forms of the two stresses occurring at different crop stages. Under natural conditions, both of these stresses are expected to occur at different levels in the topographic sequence. However, in rainfed rice ecosystems, these two stresses are often observed to occur in the same area within a growing season. This combination of stresses is especially relevant in South and Southeast Asia where rainfed rice ecosystems are major natural disaster hotspots prone to drought and flood risks (Dilley et al. 2005). For example, in eastern India, the onset of the monsoon may bring heavy rains in July–August that cause flash floods at the vegetative stage of the rice crop. However, early withdrawal of the monsoon or prolonged dry phases at the reproductive stage may cause considerable yield loss due to drought. Similarly, in the Mekong River basin in Southeast Asia, rainfed areas in countries such as Lao PDR, Thailand, Vietnam, and Cambodia are prone to submergence and drought within or across seasons and locations. Among these countries, Lao PDR is likely the most affected by drought and flood. The rainfed lowland ecosystem dominates rice cultivation in the country and is highly prone to natural disasters. In 2004, rainfed lowlands accounted for about 75% of the total area and 78% of the production (www.irri.org). Although floods are a common phenomenon in this region due to the presence of the Mekong River, an increase in drought incidences has also been observed in the past two decades (Komany 2004). TDK1 is a popular rice variety that is cultivated in a large part of the rainfed lowland ecosystems in Lao PDR. A submergence-tolerant version of this variety (TDK1-Sub1) has been developed which provides considerable tolerance to flash floods; however, both TDK1 and TDK1-Sub1 are highly susceptible to drought. *SUB1* is a major quantitative trait locus (QTL) derived from landrace FR13A that provides tolerance of 2–3 weeks of complete submergence (Septiningsih et al. 2014). This QTL was found to account for 69% of the phenotypic variance in the original identification study (Xu and Mackill 1996) and has been used extensively in marker-assisted backcross breeding (MABB) programs to improve mega-varieties with tolerance of submergence (Neeraja et al. 2007; Septiningsih et al. 2014; Toledo et al. 2015).

Similar to the advances made in submergence tolerance, considerable progress has also been made in understanding the genetics of grain yield under drought conditions in the past decade at the International Rice Research Institute (IRRI). Selection for grain yield under drought conditions has been used as an effective breeding strategy (Kumar et al. 2009; 2013; 2014; Dixit et al. 2014b). Several large-effect QTLs have also been reported for the trait in studies conducted on a wide range of mapping populations (Bernier et al. 2007; Venuprasad et al. 2009; Vikram et al. 2011; Ghimire et al. 2012; Swamy et al. 2013; Yadav et al. 2013; Mishra et al. 2013; Palanog et al. 2014; Sandhu et al. 2010; Dixit et al. 2014a, c, b). In addition to explaining a large proportion of the phenotypic variances for grain yield under drought, many of these QTLs have shown consistency of effect across genetic backgrounds. Further, studies targeting more detailed analysis of these QTLs to understand epistatic effects and QTL physiology or for fine mapping and candidate gene identification of some of these QTLs have also been conducted (Henry et al. 2014; Dixit et al. 2012a, b; 2015a). Three such QTLs, namely, *qDTY*_*3.1*_, *qDTY*_*6.1*_, and *qDTY*_*6.2*_, were identified in the background of TDK1 with large effects on grain yield under drought conditions (Dixit et al. 2014c). *qDTY*_*3.1*_ was identified as the most consistent QTL, with an effect across two levels of stress under lowland conditions and in upland mild stress conditions, and *qDTY*_*6.1*_ and *qDTY*_*6.2*_ showed effects under lowland severe stress and upland mild stress conditions (Dixit et al. 2014c).

The initial aim of this study was to pyramid *qDTY*_*3.1*_, *qDTY*_*6.1*_, and *qDTY*_*6.2*_ with *SUB1* to develop high-yielding drought- and submergence-tolerant near-isogenic lines (NILs) of TDK1 with preferred grain quality. We report a tandem selection approach combining MABB and phenotypic selection that not only allowed us to combine the four QTLs (3 drought QTLs and Sub1) together in TDK1 but also allowed us to identify lines with high yield and preferred grain quality. We also report the effect of different combinations of *DTY* QTLs on yield and yield-related traits under drought stress and non-stress conditions. The NILs identified from this study will be advanced further and tested in Lao PDR and other countries of Southeast Asia for possible release and distribution in rainfed areas as a replacement for TDK1.

## Material and methods

This study was conducted at the Ziegler Experiment Station of the IRRI, Los Baños, Laguna, Philippines. Marker-assisted backcrossing, advancement, and fixing of lines were carried out from 2012 to 2016. The drought stress, non-stress, and submergence experiments for NILs were conducted in the dry season (DS) and wet season (WS) of 2015.

### Plant material

*qDTY*_*3.1*_, *qDTY*_*6.1*_, and *qDTY*_*6.2*_ were identified in a BC_1_F_3:5_ population derived from the cross of IR55419–04 and TDK1 (Dixit et al. 2014c). TDK1 is a popular *indica* variety that covers a large area under the rainfed ecosystem in Lao PDR. This variety was developed from the cross SPT 7149–429-3/IR13423–10–2-3. It is a long-duration variety with high tillering and yield potential, but it shows high susceptibility to drought and submergence. In comparison, IR55419–04 is a drought-tolerant line developed from the cross IR12979–24-1/UPLRi 5. It is a medium-duration *indica* line with high yield potential and tolerance of drought. Lines from the mapping population showing the presence of the full segment of the three QTLs were used as the donors for the backcross program. TDK1-Sub1, the submergence-tolerant version of TDK1, was used in the backcross program as a recurrent parent to pyramid the three drought QTLs with *SUB1*.

### Genotypic data and development of chromosome maps

Rice simple-sequence repeat (SSR) markers were used for foreground selection throughout the crossing program while background recovery check at the final stage of the program was done using singlenucleotide polymorphism (SNP) markers. The SSR markers closely linked to the QTLs are given in Electronic Supplementary Material (ESM) Table 1, and the SSR genotyping protocol is presented in ESM file 1. SNP genotyping was conducted using the Infinium 6 K SNP genotyping platform (Illumina Inc., San Diego, CA) for the selected NILs. Gamma-glutamyltransferase 2 (GGT2) (Van Berloo 2008) was used for the construction of chromosome maps of NILs. SNP data also provided information on polymorphic SNPs within each QTL region that can be used for marker-assisted selection (MAS) in future studies. A list of these SNPs is provided in ESM file 2.

### QTL introgression and selection

The crossing scheme used to pyramid *qDTY*_*3.1*_, *qDTY*_*6.1*_, *qDTY*_*6.2*_, and *SUB1* into TDK1 is given in ESM Fig. 1. Seventeen BC_1_F_5_ lines with full segments of the three QTLs were selected from the mapping population and crossed to TDK1-Sub1 to develop a large BC_2_F_1_ population. A total of 658 BC_2_F_1_ plants were genotyped with foreground markers, and plants segregating for the three QTLs were selected. These plants were used to develop a large BC_2_F_2_ population (approx. 10,000 plants). From this stage onward, a tandem marker-assisted and phenotypic selection approach was used to advance the lines. First, the BC_2_F_2_ population underwent phenotypic selection for TDK1 plant type, resulting in the selection of 5700 plants. The selected plants were then genotyped with the foreground markers, and plants with different combinations of QTLs and *SUB1* were identified. A BC_2_F_3_ population of 834 lines was developed from the selected plants for further purification and testing. This population was screened under drought stress and non-stress conditions, and a total of 228 single plants were selected and advanced to the BC_2_F_4_ generation. These selected BC_2_F_4_ lines were tested again under severe drought stress conditions, and further selections were conducted. A total of 44 lines were advanced to the BC_2_F_5_ generation through single-plant selections for seed multiplication and estimation of yield potential under non-stress conditions. In addition, plant selections were conducted from all 44 lines to generate BC_2_F_6_ pure lines for screening under drought, submergence, and non-stress conditions. A total of 105 panicles were also selected from this population based on their similarity to TDK1 or TDK1-Sub1; these were used to develop a parallel set of 105 BC_2_F_6_ with grain type similar to that of the recipients. A combined set of 42 BC_2_F_7_ lines selected from this set and ten BC_2_F_7_ lines selected from the first set was then screened under drought stress and non-stress conditions. Twenty-nine BC_2_F_8_ lines were selected, and these lines underwent a final testing in replicated trials for yield under drought and non-stress conditions and for submergence tolerance. A set of ten lines was identified from this trial to test further in Lao PDR. Since the major target in this study was to develop high-yielding, drought-tolerant lines with different combinations of *DTY* QTLs and *SUB1* and with grain quality similar to that of TDK1, the lines were selected based on the foreground markers throughout the selection process. However, to estimate the recovery of the recipient genome in selected lines obtained through MAS and phenotyping results, background genotyping was conducted on selected BC_2_F_6_ lines using SNP markers (4606 SNPs). All lines showed > 90% recovery of the TDK1 genome (ESM Fig. 2). However, higher background similarity could have been achieved if the selection focus would not be overall phenotypic performance after QTL introgression.

### Management of drought and non-stress experiments

Near-isogenic lines selected at various stages of the backcross program were screened under reproductive-stage drought stress in a rainout shelter facility, while non-stress experiments were conducted in open fields at IRRI. All trials were conducted in the lowland transplanted ecosystem. In the DS of 2015 (DS-2015), the lines were planted in an alpha lattice design with two replications and a plot size of 0.7 m^2^ for the drought stress experiment, and in a single replication in an augmented randomized complete block design with a plot size of 1 m^2^ for the non-stress experiment. In the WS of 2015 (WS-2015), both the stress and non-stress experiments were conducted in an alpha lattice design in two replications. The plot size was 1.4 and 2.0 m^2^ under stress and non-stress conditions, respectively. Smaller plot sizes were maintained due to the limited amount of seeds harvested from plant selections in the previous season. In DS-2016, both stress and non-stress trials were planted in an alpha lattice design with two replications and plot sizes of 2.9 and 4 m^2^, respectively. Field and crop management was conducted as outlined by Dixit et al. (2014c).

In all experiments, days to flowering (DTF), plant height at maturity (PH), and grain yield (GY) were recorded (see Dixit et al. 2014c for the detailed procedure). Grain length and width for ten whole and de-hulled grains were measured using a Vernier caliper and the length–width ratio calculated. Kernel shape was determined using the length–width ratio as described in the standard evaluation system for rice (IRRI 2014). There were 100 grains for each NIL, and TDK1 and TDK1-Sub1 grains were sampled randomly from the seed lot and weighed to determine the grain weight.

### Management of submergence experiments

Submergence screening was conducted at seedling stage in DS-2015 and WS-2015. Selected NILs were planted in nursery beds along with TDK1 and TDK1-Sub1 and the susceptible check IR42. The lines were allowed to grow without submergence up to 14 days and then were submerged. The fields were drained after 13 days based on the survival of the susceptible check IR42. Final recovery was recorded at 7 days after draining. The lines were scored based on the standard evaluation system for rice for submergence tolerance on a scale of 1–9 in increasing order of susceptibility. Submergence screening in DS-2016 was conducted in a concrete tank facility. The lines were seeded in trays and submerged at 14 days after sowing (DAS) along with the susceptible check. The lines were de-submerged after 16 days based on the survival of the susceptible check IR42. The actual number of seedlings per line was recorded before de-submergence and 2, 7, 14, and 21 days after de-submergence, and the percentage survival was calculated.

### Statistical analysis

The data of all experiments were analyzed using CROPSTAT version 7.2.3 (http://archive.irri.org/science/software/cropstat.asp). Mixed model analysis of the data was carried out using the model

$$ {y}_{ijk}=\mu +{g}_i+{r}_j+{b}_{lj}+{e}_{ijk} $$

where *μ* is the overall mean, *g*_*i*_ is the effect of the i-th genotype, ${r}_{k_j}$ is the effect of the j-th replicate, *b*_*lk*_ is the effect of the l-th block within the j-th replicate, and *e*_*ijk*_ is the error. Genotype effects were considered to be fixed and the replicates and block effects to be random.

## Results

### Phenotypic variance

The phenotypic variance observed among the parents and progenies in field experiments and the submergence experiment is summarized in Table 1. Significant variance for all traits was observed under drought stress and non-stress conditions except for PH in DS-2015 (Table 1). IR55419–04 showed higher tolerance and significantly high GY under drought conditions in all experiments compared with TDK1 and TDK1-Sub1. TDK 1 and TDK1-Sub1 outyielded IR55419–04 in DS-2015 and DS-2016 in the non-stress experiment. In WS-2015, an opposite pattern of yield under non-stress conditions was observed, in which IR55419–04 showed higher yield than the other two parents (Table 1); the difference was non-significant in DS-2015 and WS-2015, while a significant difference was observed in DS-2016 for the trait. The mean yield of the progenies remained between the high- and low-yielding parents in all experiments. IR55419–04 was the early-flowering parent among the three, while TDK1 and TDK1-Sub1 showed a similar flowering time under both stress and non-stress conditions. The progeny means lay between IR55419–04 and the two late-flowering parents. All three parents showed similar PH in all experiments, except in DS-2016 when IR55419–04 had lower PH than the other two parents under the non-stress conditions. However, a higher reduction in PH was observed for TDK1 and TDK1-Sub1 under the stress conditions. The mean PH of the progenies was also similar to that of the recipient parents under drought stress and non-stress conditions. TDK1 and IR55419–04 showed high susceptibility to submergence compared with the tolerant donor TDK1-Sub1; the progeny mean fell between those of the tolerant parent and the two susceptible parents (Table 1).

**Table 1:** Analysis of variance in the drought stress, non-stress, and submergence experiments using mean values of parents and progenies

Designation^ab^	Grain yield (kg ha^−1^)						Days to flowering						Plant height (cm)						Submergence experiment	
	DS-2015		WS-2015		DS-2016		DS-2015		WS-2015		DS-2016		DS-2015		WS-2015		DS-2016		DS-2015 (score)^b^	DS-2016 (% survival)
	S	NS	S	NS	S	NS	S	NS	S	NS	S	NS	S	NS	S	NS	S	NS		
TDK1	14	6449	2	3636	48	6415	108	98	123	107		88	65	88	119	119	69	103	9	0
TDK1-Sub1	0	6146	2	4125	0	6110		101	122	105	104	87	61	90	116	116	75	100	3	5
IR55419–04	882	5254	449	4415	1032	4227	72	81	80	83	69	71	68	89	115	115	88	90	9	0
Progenies	346	6144	125	4855	210	4854	95	90	97	88	86	80	64	91	92	121	77	102	6	14
*P* value^c^	****	**	****	**	****	****	****	****	****	****	****	****		****	***	****	****	****	****	****
Standard error of difference	166	937	81	629	206	394	3	9	6	2	1	5	9	4	8	6	3	6	1	10

DS, Dry season; WS, wet season; 2015, 2016, years of trials; S, stress experiment; NS, non-stress experiment

^a^See sections Plant material and QTL introgression and selection for a description of the parents and progenies

^b^As a measure of tolerance of submergence, the lines were scored on a scale of 1–9, with 1 indicating a low susceptibility (high tolerance) to submergence and 9 indicating a high susceptibility to submergence

^c^ *P* is the probability of difference between genotypes, where **, ***, **** indicates significance at the 1, 0.1, and 0.01% *P* levels, respectively

### Trait correlations and QTL class analysis

The phenotypic correlations between GY, PH, and DTF under drought stress and non-stress conditions in DS-2015 are presented in Table 2. GY under stress conditions showed significant negative correlations with DTF, thereby revealing the advantage of early-flowering lines under stress conditions. However, a positive correlation between GY and DTF was observed under non-stress conditions. Apart from this, a significant negative correlation between stress and non-stress in GY was also observed. No significant correlations were observed between GY and PH under drought conditions; however, under non-stress conditions, the two traits were positively correlated.

**Table 2:** Phenotypic correlations between traits under drought stress and non-stress conditions in DS-2015

Traits	DTF-NS	PHN-S	GY-NS	DTF-S	PH-S	GY-S
DTF-NS	1					
PH-NS	− 0.12	1				
GY-NS	0.31*	0.50**	1			
DTF-S	0.84**	− 0.18	0.31*	1		
PH-S	− 0.14	0.36*	0.07	− 0.20	1	
GY-S	− 0.74**	0.08	− 0.31*	− 0.88**	0.13	1

*, **Significant at the 5 and 1% levels of significance, respectively

DTF, Days to flowering; PH, plant height at maturity; GY, grain yield

**Fig. 1:**
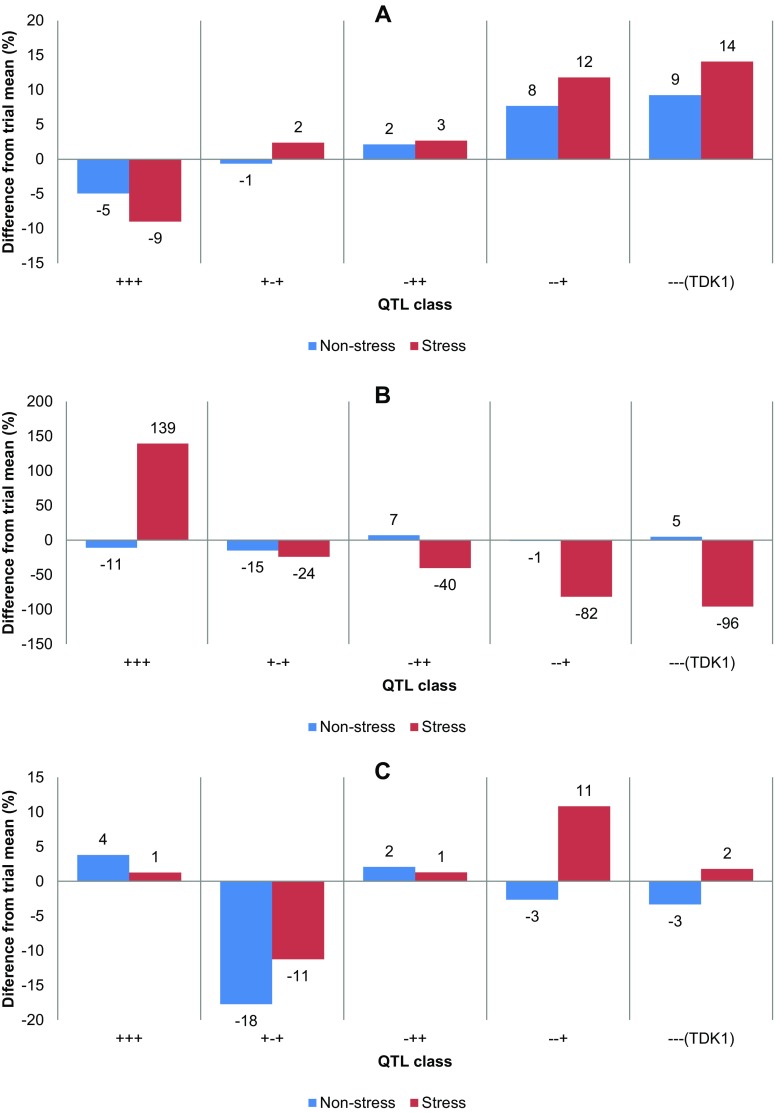
Effect of combinations of *qDTY*_*3.1*_, *qDTY*_*6.1*_, and *qDTY*_*6.2*_, 3 quantitative trait loci (QTLs) identified in the background of rice variety TDK1 with large effects on grain yield under drought conditions, on a days to flowering, b grain yield (GY) and c plant height under drought stress and non-stress conditions. +++ class refers to lines with all 3 QTLs, +−+ class refers to lines with *qDTY*_*3.1*_ and *qDTY*_*6.2*_, −++ class refers to lines with *qDTY*_*6.1*_ and *qDTY*_*6.2*_, −−+ class refers to lines with *qDTY*_*6.2*_ only; TDK1 (−−−) is taken as the baseline for lines without any QTLs. Lines with three QTLs showed the highest yield advantage under drought conditions and earlier flowering and maintained plant height. *qDTY*_*3.1*_ led to a yield penalty under non-stress conditions

In order to understand the correlations of the three traits at the genetic level and to understand the effect of the three *DTY* on these traits, a QTL class analysis was conducted with *qDTY*_*3.1*_, *qDTY*_*6.1*_, and *qDTY*_*6.2*_ (Fig. 1). The class analysis revealed an effect of *qDTY*_*3.1*_ on DTF of the lines (Fig. 1a). The two classes of lines with this QTL [+++ (possessing all 3 QTLs) and +−+ (possessing *qDTY*_*3.1*_ and *qDTY*_*6.2*_] showed early flowering compared with the two without them [−++ (possessing *qDTY*_*6.1*_ and *qDTY*_*6.2*_) and −−+ (with *qDTY*_*6.2*_ only)]. *qDTY*_*6.1*_ was also observed to play a role along with *qDTY*_*3.1*_ in causing earliness in the lines. It was observed that lines with both these QTLs (+++) flowered much earlier than those with one of the two QTLs (+−+ and −++) and that the lines without the two QTLs [−−+ (possessing *qDTY*_*6.2*_ only) flowered even later. TDK1 [−−−, without any of the 3 QTLs) flowered the latest of all classes. For GY (Fig. 1b), a similar relation was observed under drought conditions, for which lines with both *qDTY*_*3.1*_ and *qDTY*_*6.1*_ (+++) showed higher GY than those with one of the two QTLs (−++ and +−+). *qDTY*_*3.1*_, however, had a larger effect on GY than *qDTY*_*6.1*_, as a smaller difference from the trial mean was observed for class +−+ than for class −++. These two classes were followed by the last class with *qDTY*_*6.2*_ only (−−+), whereas all classes yielded higher GY under drought conditions than TDK1 (−−−). *qDTY*_*3.1*_ caused a reduction in GY under non-stress conditions as the QTL classes +++ and +−+ showed lower mean yields than the trial mean. In the case of PH, *qDTY*_*6.1*_ seemed to be the only QTL showing an effect under non-stress conditions as classes +++ and −++ consistently showed positive differences from the trial mean (Fig. 1c). No clear pattern of effect of the other two QTLs was observed for PH.

### Selected lines, their performance, and grain type

**Fig. 2:**
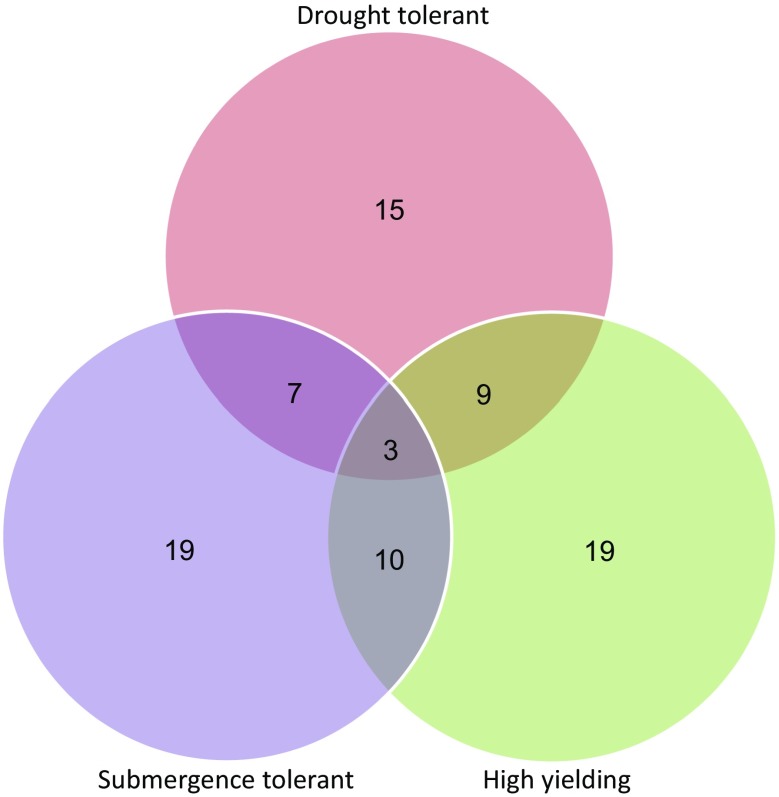
Vann diagram showing the proportion of lines with tolerance of drought and submergence and with high yield from the set of lines screened in the dry season of 2015. Numbers in the circles refer to lines with one of the three traits while those in the intersecting areas show the lines with two or all three traits. In total, 29 lines showed the presence of a combination of two or three traits

These results showed the advantage of *qDTY*_*3.1*_ under stress conditions and its disadvantage under non-stress conditions. Apart from this, the program also targeted the identification of lines tolerant of submergence and drought and maintenance of TDK1/TDK1-Sub1 grain quality. Because of this, phenotypic selection at the end of the MAS program became very important to identify high-yielding drought-tolerant lines. The identified lines screened under drought stress and non-stress conditions showed varying performance for the two stresses and GY under non-stress conditions. Of the 44 lines screened, 15 lines showed high GY under drought conditions; however, only nine of these 15 lines showed high GY under non-stress conditions and only seven of these same 15 lines showed tolerance of both drought and submergence (Fig. 2). Similarly, of the 19 lines with high GY potential under non-stress conditions, ten showed tolerance of submergence. We identified three lines in which all three traits were successfully combined. These lines showed increased tolerance of submergence along with high GY under drought conditions compared with TDK1 and more than/similar GY as TDK1 under non-stress conditions. The performance of seven selected lines is shown in Table 3. Four of these lines showed high GY under drought stress and non-stress conditions compared with TDK1; however, these lines showed similar susceptibility to submergence as TDK1. The other three lines showed higher tolerance of both stresses than TDK1 and high GY. In DS-2015, the mean GY of six of the seven lines ranged between the mean of the tolerant parent IR55419–04 and that of the susceptible parent TDK1. However, IR102776–31–66-2-2-2 showed higher GY than the tolerant parent IR55419–04. In WS-2015, a much more severe stress was imposed than in DS-2015. A similar pattern of GY was observed for the lines, with mean yields ranging between those of the tolerant and susceptible parents. Although the differences in GY became less apparent in this season due to the high severity of stress, three of the lines still showed significantly higher GY than TDK1. The two susceptible parents showed almost no GY in both seasons (Table 3). A second set of lines was also identified from DS-2015 screening (ESM Table 2). These lines were those that showed relatively moderate GY under drought stress conditions compared with the first set of lines, but they had a grain type much closer to that of TDK1-Sub1.

**Table 3:** Selected near-isogenic lines and their grain yield, days to 50% flowering, and plant height under drought stress and non-stress conditions in DS-2015 and WS-2015 and under submergence conditions in DS-2015

Line	DS-2015						WS-2015					Submergence	QTL	
	GY (kg ha^−1^)		DTF		PH (cm)		GY (kg ha^−1^)		DTF	PH (cm)				
	NS	S	NS	S	NS	S	NS	S	NS	S	NS	S	B	L
IR102774–11–128-1-4-3	6773	466	86	88	98	73	5244	182	87	84	129	101	9	H++
IR102776–37–52–1-1-3	6760	534	86	89	105	81	5050	300	86	82	132	106	9	−++
IR102775–24–97-1-1-1	6448	904	90	87	84	70	4305	95	87	96	107	78	9	+++
IR102777–5–64-4-1-5	6656	595	87	88	84	60	4144	238	86	88	108	91	9	+H-
IR102776–31–66-2-2-2	6615	1173	87	87	94	65	5533	58	88	93	117	80	6	+++
IR102774–15–32–3-1-2	7255	718	87	91	110	66	5511	40	84	91	132	104	5	+++
IR102777–5–83–1-2-2	7112	519	87	89	101	82	4897	37	90	104	132	92	4	−++
IR55419–04	5254	882	81	72	89	68	4415	449	83	80	115	100	9	
TDK1	6449	14	98	108	88	65	3636	2	107	123	119	80	9	
TDK1-Sub1	6146	4	101		90	61	4125	2	105	122	116	83	3	

Grain measurements were conducted for all the identified lines. In general, the grain shape of all lines was close to each other. and a majority of them, including the recurrent parent TDK1 and TDK1-Sub1, were classified as slender grain types (ESM Table 3a). However, IR102774–11–128-1-4-3 showed medium grain type that was similar to that of the donor parent IR55419–04. All lines with *SUB1* showed a similar light-colored hull as TDK1-Sub1, whereas those without *SUB1* showed a similar hull color as TDK1. An exception to this was line IR102777–5–64-4-1-5, which had a white hull color despite lacking *SUB1*; this can be attributed to an effect from the donor line IR55419–04, which also has light hull color. All seven lines selected based on GY, tolerance of drought and submergence, and QTL presence were similar to the recipient in terms of grain measurements. They all showed intermediate chalkiness between TDK1 and IR55419–04 (ESM Fig. 3a). However, the second set of lines was much closer to the recipient parent in terms of grain shape and waxiness (ESM Table 3b; ESM Fig. 3b). During the MABB program, we also created pure lines based on panicle selection conducted for grain type. These were tested in DS-2016 in large plots along with the previously identified lines. The performance of these lines is presented in ESM Table 4. We identified a total of seven lines from this set that showed tolerance of drought and submergence. Consistent with previous seasons, IR102776–31–66-2-2-2 showed high yield under drought conditions and tolerance of submergence (ESM Table 4). All lines showed similar grain structure as the recipient, and IR102777–18–128-2-1-4 also had high waxiness.

## Discussion

Our study reports the development of drought- and submergence-tolerant versions of a popular rice variety, TDK1, from Lao PDR and the effect of different combinations of *DTY* QTLs on the performance of lines under drought and non-stress conditions. NILs of TDK1 with different combinations of *qDTY*_*3.1*_ *, qDTY*_*6.1*_ *,* and *qDTY*_*6.2*_ and *SUB1* were developed and tested under drought stress, non-stress, and submergence conditions to identify the best NILs and QTL combinations. The study also aimed at determining the effect of combining the *DTY* QTLs with *SUB1* on the performance of lines. *qDTY*_*3.1*_, *qDTY*_*6.1*_, and *qDTY*_*6.2*_ were identified in a BC_1_-derived population developed from the cross of IR55419–04 and TDK1 (Dixit et al. 2014c). We crossed lines from this mapping population to TDK1-Sub1 to develop a BC_2_ population segregating for all three *DTY* QTLs and *SUB1*. Maintenance of grain quality, plant type, and yield potential is of the utmost importance while targeting the development of a product for commercial release and cultivation. In this case, it was required to develop lines with the same or higher grain quality and GY than the recipient variety TDK1 or TDK1-Sub1. Apart from this, the QTL with the largest effect on GY under drought (*qDTY*_*3.1*_) targeted in this study is known to reduce GY under non-stress conditions (Venuprasad et al. 2009). In order to eliminate any undesirable effects on other traits and develop high-yielding lines with TDK1 or TDK1-Sub1 grain type, we applied a tandem selection approach using both genotypic data and phenotypic selection to advance the lines. A similar approach has been described by Han et al. (1997) in barley. However, in our case, we targeted phenotypic selection for different traits at different stages of the MAS to maximize the genetic gain (ESM Fig. 1). The screening under drought stress, non-stress, and submergence conditions revealed that there was high variation among the parents and progenies (Table 1), which allowed the selection of lines with all/most of the desired characters. This variation may have resulted from positive interaction between QTLs or interaction of one or more QTLs with the genetic background (Dixit et al. 2014c). The analysis of the effect of *DTY* QTLs on GY, DTF, and PH showed a high positive effect of *qDTY*_*3.1*_ on GY under drought conditions, whereas a negative effect of this QTL was observed under non-stress conditions (Fig.1). A similar response of this QTL has been reported previously in studies in which this QTL was identified (Venuprasad et al. 2009; Dixit et al. 2014c). Apart from this, similar to Dixit et al. ( 2014c), a complementary effect of *qDTY*_*3.1*_ and *qDTY*_*6.1*_ was observed in this study. and *qDTY*_*6.2*_ showed a relatively smaller effect. The similarity and consistency of the effect patterns of these two QTLs in the original mapping population and NIL trials not only show their suitability for MABB but also show that the presence of the *SUB1* gene does not alter the effect pattern of *DTY* QTLs in this specific case. Despite the overall negative effect of *qDTY*_*3.1*_ on GY under non-stress conditions, we were able to identify NILs with yield potential similar to or more than TDK1 and TDK1-Sub1. A total of seven high-yielding NILs were ultimately identified after the first two seasons of screening (Table 3). Four of these showed higher tolerance of drought while the other three showed tolerance of drought and submergence compared with the parent TDK1 (Table 3). The association of RM468, one of the foreground markers of this QTL, has been reported previously to have a negative effect on GY under non-stress conditions (Dixit et al. 2014c). In our study, of the 44 NILs tested under drought stress and non-stress conditions at the end of the MAS program, two high-yielding NILs with a full segment of *qDTY*_*3.1*_ were identified. This result suggests a recombination event leading to the elimination of the factors causing the negative effect on GY due to continuous phenotypic selection coupled with MAS. In terms of grain quality, two sets of NILs were identified: the first set included those with intermediate waxiness (ESM Fig.  3a), while the second included those with high waxiness similar to that of TDK1 and TDK1-Sub1 (ESM Fig. 3b). All except one of the identified NILs showed similar grain shape as TDK1 and TDK1-Sub1 (ESM Table 3; ESM Fig. 3). TDK1 has a dark-colored hull and TDK1-Sub1 has a light-colored hull. This variation in hull color has also been observed in other cases, such as for the Indian rice variety Swarna, for which the *SUB1* introgression has led to a light-colored hull. In our study, all lines with *SUB1* showed similar light-colored hull as TDK1-Sub1, whereas the majority of those without *SUB1* showed a similar hull color as TDK1. These features of the two sets of NILs are very important to identify their potential use and target environment for release as varieties. While the first set can be a suitable breeding material for high tolerance of drought and submergence and further increasing waxiness, the second set of NILs can be suitable for release in drought- and submergence-prone areas where waxiness is preferred. Among all lines evaluated, IR102776–31–66-2-2-2 looks to be outperforming other lines, as was also confirmed in WS-2016 experiments in drought and non-stress conditions.

MAS has been used extensively in rice breeding to pyramid genes/QTLs of interest in popular rice cultivars. Several studies have targeted genes conferring tolerance of major biotic stresses to successfully develop tolerant lines through MAS (Huang et al. 1997; Hittalmani et al. 2000; Singh et al. 2001). Similarly, QTLs/genes underlying GY-related traits and grain quality in rice have also been used for MAS (Joseph et al. 2004; Zhang et al. 2006; Yi et al. 2009). For abiotic stress tolerance in rice, some famous examples of successful MAS are of the submergence tolerance genes *SUB1* (Neeraja et al. 2007) and *SNORKEL 1* (Hattori et al. 2009), *Saltol* for salinity tolerance (Linh et al. 2012), *DTY* QTLs (Swamy et al. 2013; Dixit et al. 2015a, b; Shamsudin et al. 2016a, b), and Dro 1 (Uga et al. 2013) for drought tolerance. However, studies on combining tolerance of two major abiotic stresses through MAS are very rare. Our study has successfully demonstrated that, through a systematic MAS program combined with phenotypic selection, not only tolerance of multiple stresses can be achieved but also other important characters such as yield potential, plant type, and grain quality can be retained. In countries such as Lao PDR, where large areas under rice cultivation suffer vegetative-stage submergence and reproductive-stage drought, these lines could prove to be an efficient tool to ensure yield stability. These lines can also serve as valuable genetic material to be used for further breeding of high-yielding drought- and submergence-tolerant varieties in local breeding programs.

## Electronic supplementary material

Supplementary Table 1 Details of rice microsatellite markers linked to *qDTY*_*3.1*_ *, qDTY*_*6.1*_, *qDTY*_*6.2*_ and Sub 1. Physical positions (bp) of the markers was downloaded from www.gramene.org and https://blast.ncbi.nlm.nih.gov with Nipponbare as the reference genome. Those in bold are closely linked peak markers. (DOCX 25.7 kb)

Supplementary Table 2 Performance of NILs with moderate yield advantage under drought stress and high similarity to grain type of recipient parent in DS2015. (DOCX 23.6 kb)

Supplementary Table 3 Grain type variations observed in two sets of selected lines and parents:** a** grain type of lines with high yield under drought stress and non-stress;** b** grain parameters of lines with intermediate yield under drought but high grain similarity to the recipient parents. (DOCX 27 kb)

Supplementary Table 4 Performance of pure lines developed through panicle selection under drought and submergence along with their grain parameters. (DOCX 25.3 kb)

Supplementary Figure 1 Marker-assisted breeding scheme used to develop drought- and submergence-tolerant NILs of recipient parent TDK1. BC (followed by the number as subscript) refers to the backcross generation; F (followed by the number as subscript) refers to the filial generation developed through selfing after the backcross. Numbers within parentheses after each generation refer to the number of plants generated. The strategy shows the coupling of MAS with phenotypic selection to achieve maximum advantage by combining major QTLs (through MAS) and minor favorable alleles (through phenotypic selection). (PDF 19.5 kb)

Supplementary Figure 2 Graphical genotype of one of the high-yielding, drought-tolerant NILs generated through Infinium 6 K SNP genotyping. Light-gray color shows recipient (TDK1) allele, red color shows donor (IR55419–04) allele, blue color shows the introgressed *DTY* QTL regions, green color shows the *SUB1* region, and dark gray lines show heterozygotes. Selection focus on presence of QTLs and overall phenotypic performance led to relatively lower background clarity but allowed selection of best performing NILs. (PDF 78.7 kb)

Supplementary Figure 3 Grain type variations obtained within the NILs compared to IR55419–04 (non- waxy) and TDK1/ TDK1 Sub1 (waxy). Waxiness ranged from intermediate to high in the NILs. Line IR102776–31–66-2-2-2 (**a**) showed intermediate waxiness while IR102777–18–128-2-1-4 had waxy grain type similar to TDK1 and TDK1 Sub 1. (PDF 1859 kb)

Supplementary File 1 SSR genotyping protocol used for foreground selection during the NIL development process. (DOCX 19.5 kb)

Supplementary File 2 SNP markers showing polymorphism between the two parents IR55419–04 and TDK1 Sub1 within peak regions of all four QTLs. (XLSX 24.7 kb)
