# Supplementary material for: Combining drought and submergence tolerance in rice: marker-assisted breeding and QTL combination effects
Source: Mol Breed. 2017 Nov 4;37(12):143. doi: 10.1007/s11032-017-0737-2 (PMC5670188; doi:10.1007/s11032-017-0737-2)
Supplement: Supplementary file 1 — Details of rice microsatellite markers linked to qDTY 3.1 , qDTY 6.1, qDTY 6.2 and Sub 1. Physical positions (bp) of the markers was downloaded from www.gramene.org and https://blast.ncbi.nlm.nih.gov with Nipponbare as the reference genome. Those in bold are closely linked peak markers. (DOCX 25.7 kb) [file 11032_2017_737_MOESM1_ESM.docx]

**Supplementary Table 1:** Details of rice microsatellite markers linked to *qDTY_3.1_, qDTY_6.1_*, *qDTY_6.2_* and Sub 1. Physical positions (bp) of the markers was downloaded from [www.gramene.org](http://www.gramene.org) and <https://blast.ncbi.nlm.nih.gov> with Nipponbare as the reference genome. Those in bold are closely linked peak markers.

| **QTL** | **Marker** | **Forward primer** | **Reverse primer** | **PCR product size (bp)** | **Chr** | **SSR start**  **(bp)** | **SSR end**  **(bp)** |
| --- | --- | --- | --- | --- | --- | --- | --- |
| *qDTY_3.1_* | RM168 | TGCTGCTTGCCTGCTTCCTTT | GAAACGAATCAATCCACGGC | 116 | 3 | 28091534 | 28091737 |
|  | **RM186** | **TCCTCCATCTCCTCCGCTCCCG** | **GGGCGTGGTGGCCTTCTTCGTC** | **124** | **3** | **28806696** | **28806820** |
|  | **RM55** | **CCGTCGCCGTAGTAGAGAAG** | **TCCCGGTTATTTTAAGGCG** | **226** | **3** | **29052279** | **29052297** |
|  | **RM293** | **TCGTTGGGAGGTATGGTACC** | **CTTTATCTGATCCTTGGGAAGG** | **207** | **3** | **31657113** | **31657132** |
|  | RM468 | CCCTTCCTTGTTGTGGCTAC | TGATTTCTGAGAGCCAACCC | 265 | 3 | 32674852 | 32675116 |
| *qDTY_6.1_* | RM508 | GGATAGATCATGTGTGGGGG | ACCCGTGAACCACAAAGAAC | 235 | 6 | 441616 | 441850 |
|  | **RM586** | **ACCTCGCGTTATTAGGTACCC** | **GAGATACGCCAACGAGATACC** | **271** | **6** | **1476793** | **1477087** |
|  | **RM587** | **ACGCGAACAAATTAACAGCC** | **CTTTGCTACCAGTAGATCCAGC** | **217** | **6** | **2291804** | **2292076** |
|  | RM217 | ATCGCAGCAATGCCTCGT | GGGTGTGAACAAAGACAC | 133 | 6 | 4235183 | 4235200 |
| *qDTY_6.2_* | **RM3** | **ACACTGTAGCGGCCACTG** | **CCTCCACTGCTCCACATCTT** | **145** | **6** | **19500417** | **19500434** |
|  | **RM541** | **TATAACCGACCTCAGTGCCC** | **CCTTACTCCCATGCCATGAG** | **158** | **6** | **19514538** | **19514557** |
| *SUB1* | **ART5** | **CAGGGAAAGAGATGGTGGA** | **TTGGCCCTAGGTTGTTTCAG** | **200** | **9** | **6392654** | **6392672** |
