# Supplementary material for: Combining drought and submergence tolerance in rice: marker-assisted breeding and QTL combination effects
Source: Mol Breed. 2017 Nov 4;37(12):143. doi: 10.1007/s11032-017-0737-2 (PMC5670188; doi:10.1007/s11032-017-0737-2)
Supplement: Supplementary file 2 — Performance of NILs with moderate yield advantage under drought stress and high similarity to grain type of recipient parent in DS2015. (DOCX 23.6 kb) [file 11032_2017_737_MOESM2_ESM.docx]

Supplementary table 2: Performance of NILs with moderate yield advantage under drought stress and high similarity to grain type of recipient parent in DS2015.

| **Designation** | **GYNS** | **GYS** | **DTFNS** | **DTFS** | **PHNS** | **PHS** | **7DAD** | **QTL** |
| --- | --- | --- | --- | --- | --- | --- | --- | --- |
| IR102777-18-128-2-1-3 | 5456 | 156 | 89 | 96 | 87 | 61 | 3 | +-H |
| IR102774-32-90-4-2-1 | 6853 | 135 | 93 | 102 | 95 | 60 | 4 | +HH |
| IR102774-31-26-2-3-1 | 5770 | 111 | 92 | 100 | 87 | 54 | 4 | +-- |
| IR102776-32-118-2-2-2 | 6248 | 289 | 95 | 95 | 91 | 58 | 7 | -++ |
| IR102775-26-113-2-3-3 | 6429 | 247 | 87 | 95 | 93 | 68 | 9 | -++ |
| TDK1 | 6449 | 14 | 98 | 108 | 88 | 65 | 9 |  |
| TDK1-Sub1 | 6146 | 4 | 101 |  | 90 | 61 | 3 |  |
| IR55419-04 | 5254 | 882 | 81 | 72 | 89 | 68 | 9 |  |
