# Supplementary material for: Combining drought and submergence tolerance in rice: marker-assisted breeding and QTL combination effects
Source: Mol Breed. 2017 Nov 4;37(12):143. doi: 10.1007/s11032-017-0737-2 (PMC5670188; doi:10.1007/s11032-017-0737-2)
Supplement: Supplementary file 3 — Grain type variations observed in two sets of selected lines and parents: a grain type of lines with high yield under drought stress and non-stress; b grain parameters of lines with intermediate yield under drought but high grain similarity to the recipient parents. (DOCX 27 kb) [file 11032_2017_737_MOESM3_ESM.docx]

Supplementary table 3: Grain type variations observed in two sets of selected lines and parents: (A) grain type of lines with high yield under drought stress and non-stress; (B) grain parameters of lines with intermediate yield under drought but high grain similarity to the recipient parents.

| **Subset** | **Line** | **Whole grain** | | |  | **De-hulled grain** | | | **100- grain weight (g)** | **Grain shape** | **Waxiness** |
| --- | --- | --- | --- | --- | --- | --- | --- | --- | --- | --- | --- |
|  |  | **Length (mm)** | **Width (mm)** | **L:W** |  | **Length (mm)** | **Width (mm)** | **L:W** |  |  |  |
| A | IR102774-11-128-1-4-3 | 9.4 | 3.1 | 3 |  | 6.8 | 2.6 | 2.6 | 3.1 | Medium | Non-waxy |
|  | IR102776-37-52-1-1-3 | 9.7 | 3.1 | 3.1 |  | 6.9 | 2.6 | 2.7 | 3.1 | Slender | Non-waxy |
|  | IR102775-24-97-1-1-1 | 9.8 | 2.9 | 3.4 |  | 7.2 | 2.5 | 2.9 | 2.9 | Slender | Non-waxy |
|  | IR102777-5-64-4-1-5 | 10.0 | 3.0 | 3.4 |  | 7.2 | 2.5 | 2.9 | 2.9 | Slender | Non-waxy |
|  | IR102776-31-66-2-2-2 | 10.8 | 2.9 | 3.7 |  | 7.7 | 2.4 | 3.2 | 3.4 | Slender | Non-waxy |
|  | IR102774-15-32-3-1-2 | 10.0 | 2.9 | 3.4 |  | 7.2 | 2.5 | 2.9 | 3.2 | Slender | Non-waxy |
|  | IR102777-5-83-1-2-2 | 10.5 | 3.0 | 3.5 |  | 7.6 | 2.5 | 3.0 | 3.4 | Slender | Non-waxy |
| B | IR102777-18-128-2-1-3 | 10.2 | 2.9 | 3.6 |  | 7.2 | 2.3 | 3.1 | 2.8 | Slender | Waxy |
|  | IR102774-32-90-4-2-1 | 11.1 | 2.9 | 3.8 |  | 7.8 | 2.4 | 3.3 | 3.3 | Slender | Waxy |
|  | IR102774-31-26-2-3-1 | 10.4 | 2.8 | 3.7 |  | 7.3 | 2.3 | 3.2 | 3.0 | Slender | Waxy |
|  | IR102776-32-118-2-2-2 | 10.4 | 3.0 | 3.5 |  | 7.3 | 2.4 | 3.0 | 3.1 | Slender | Waxy |
|  | IR102775-26-113-2-3-3 | 10.7 | 3.0 | 3.6 |  | 7.5 | 2.4 | 3.1 | 3.1 | Slender | Waxy |
|  | IR55419-04 | 9.1 | 3.0 | 3.0 |  | 6.5 | 2.6 | 2.5 | 2.7 | Medium | Non-waxy |
|  | TDK1-Sub1 | 10.3 | 2.8 | 3.6 |  | 7.2 | 2.4 | 3.1 | 3.0 | Slender | Waxy |
|  | TDK1 | 10.5 | 3.0 | 3.5 |  | 7.3 | 2.4 | 3.0 | 2.9 | Slender | Waxy |

L:W: length-width ratio.
