# Supplementary material for: Combining drought and submergence tolerance in rice: marker-assisted breeding and QTL combination effects
Source: Mol Breed. 2017 Nov 4;37(12):143. doi: 10.1007/s11032-017-0737-2 (PMC5670188; doi:10.1007/s11032-017-0737-2)
Supplement: Supplementary file 4 — Performance of pure lines developed through panicle selection under drought and submergence along with their grain parameters. (DOCX 25.3 kb) [file 11032_2017_737_MOESM4_ESM.docx]

Supplementary table 4: Performance of pure lines developed through panicle selection under drought and submergence along with their grain parameters.

| **Designation** | **Grain yield** | | **Days to flowering** | | **Plant height** | | **Submergence (% survival)** | **Grain shape** | **Grain type** |
| --- | --- | --- | --- | --- | --- | --- | --- | --- | --- |
|  | **GYS** | **GYNS** | **DTFS** | **DTFNS** | **HTS** | **HTNS** |  |  |  |
| IR102777-6-86-2-2-14 | 117 | 6611 | 96 | 83 | 67 | 98 | 16 | Slender | Non-waxy |
| IR102777-6-86-2-2-7 | 163 | 5314 | 89 | 80 | 67 | 95 | 44 | Slender | Non-waxy |
| IR102776-18-21-1-1-2 | 206 | 4525 | 82 | 77 | 68 | 94 | 31 | Slender | Non-waxy |
| IR102777-18-64-1-2-12 | 284 | 4592 | 78 | 79 | 68 | 96 | 28 | Slender | Non-waxy |
| IR102777-18-128-2-1-4 | 478 | 5290 | 77 | 80 | 75 | 94 | 40 | Slender | Waxy |
| IR102777-6-86-2-2-11 | 479 | 4966 | 80 | 79 | 71 | 94 | 38 | Slender | Non-waxy |
| IR102776-31-66-2-2-2 | 587 | 4646 | 74 | 77 | 59 | 92 | 29 | Slender | Non-waxy |
| Trial mean | 210 | 4854 | 86 | 80 | 77 | 102 | 14 |  |  |
| TDK1 | 48 | 6415 |  | 88 | 69 | 103 | 0 | Slender | Waxy |
| TDK1-Sub1 | 0 | 6110 | 104 | 87 | 75 | 100 | 5 | Slender | Waxy |
| IR55419-04 | 1032 | 4227 | 69 | 71 | 88 | 90 | 0 | Medium | Non waxy |
| SED | 206 | 394 | 1 | 5 | 3 | 6 | 10 |  |  |
| *P* | **** | **** | **** | **** | **** | **** | **** |  |  |
