# Supplementary figures and images for: Combining drought and submergence tolerance in rice: marker-assisted breeding and QTL combination effects
Source: Mol Breed. 2017 Nov 4;37(12):143. doi: 10.1007/s11032-017-0737-2 (PMC5670188; doi:10.1007/s11032-017-0737-2)

## Cross/generation

## Selection/screening

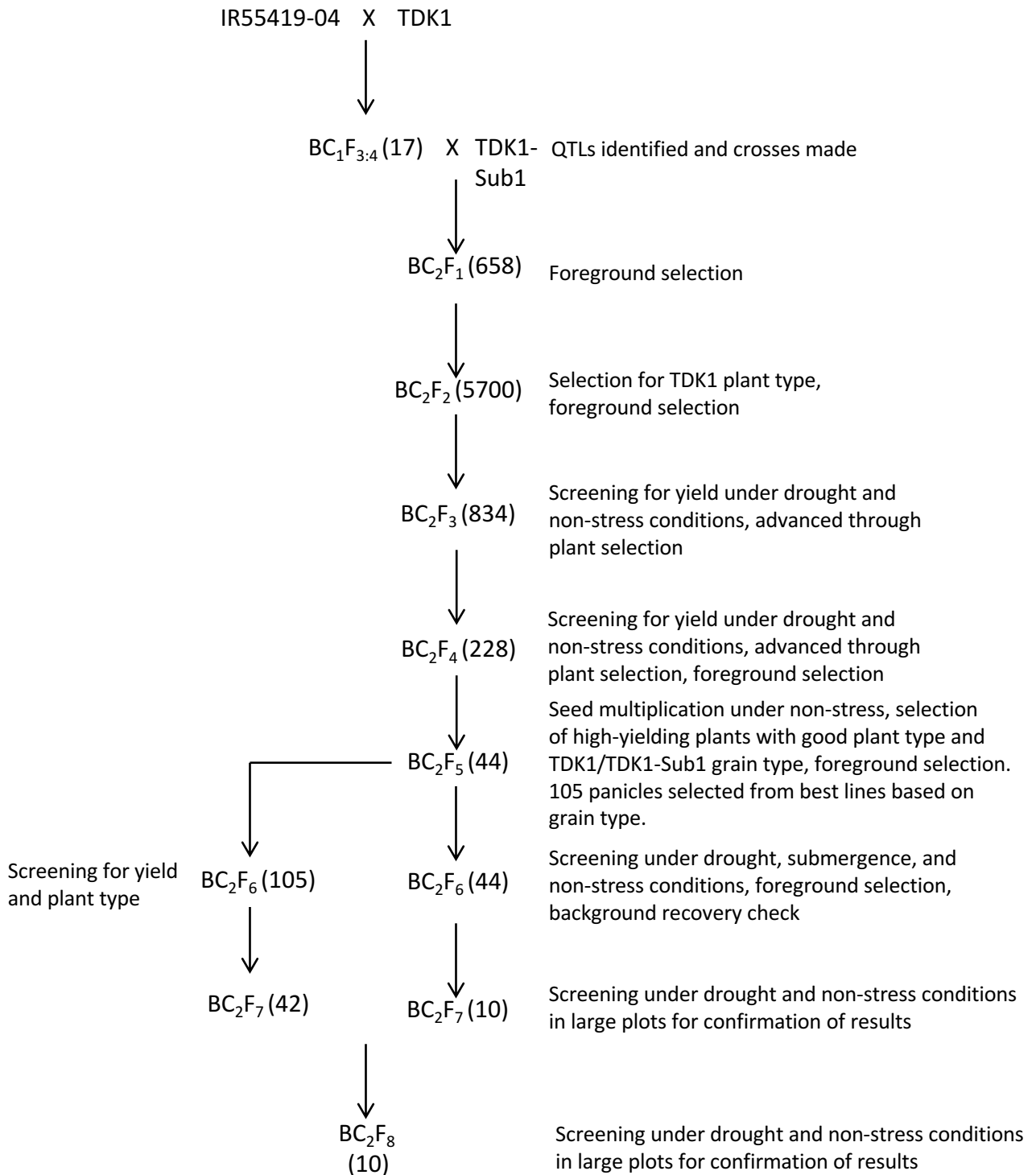

Supplement: Supplementary file 5 — Marker-assisted breeding scheme used to develop drought- and submergence-tolerant NILs of recipient parent TDK1. BC (followed by the number as subscript) refers to the backcross generation; F (followed by the number as subscript) refers to the filial generation developed through selfing after the backcross. Numbers within parentheses after each generation refer to the number of plants generated. The strategy shows the coupling of MAS with phenotypic selection to achieve maximum advantage by combining major QTLs (through MAS) and minor favorable alleles (through phenotypic selection). (PDF 19.5 kb) [file 11032_2017_737_MOESM5_ESM.pdf]

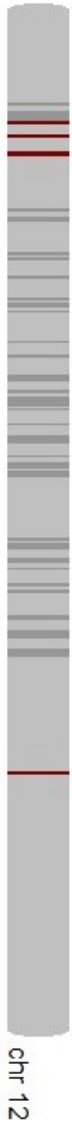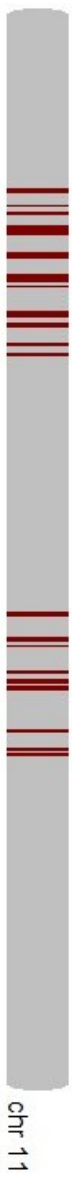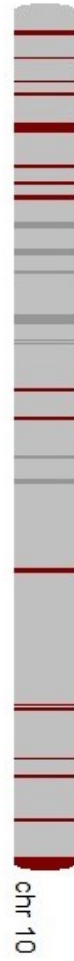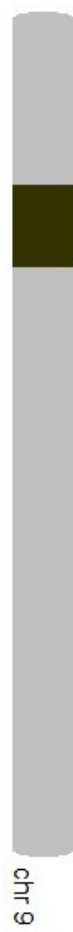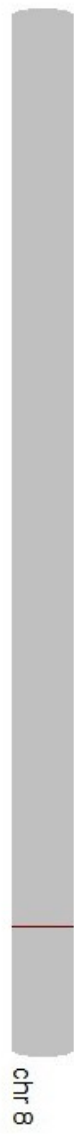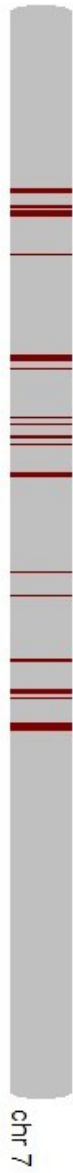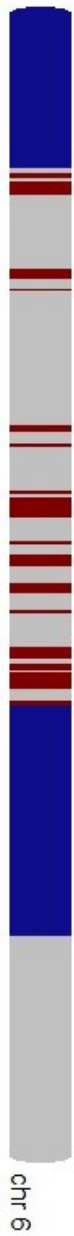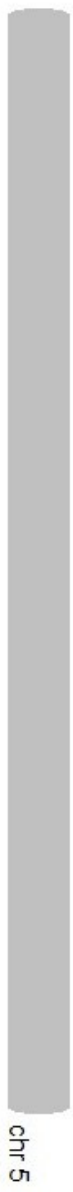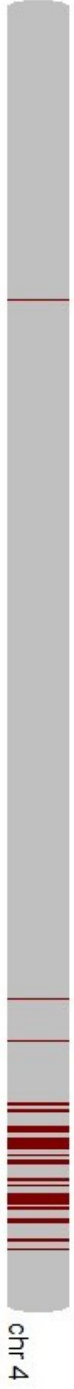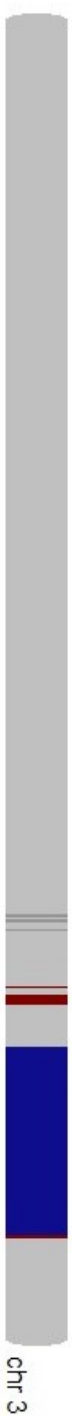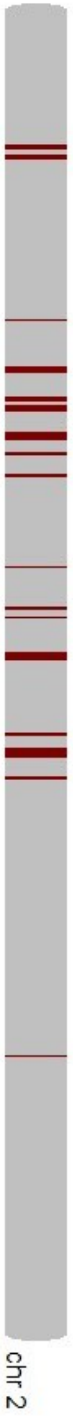

Supplement: Supplementary file 6 — Graphical genotype of one of the high-yielding, drought-tolerant NILs generated through Infinium 6 K SNP genotyping. Light-gray color shows recipient (TDK1) allele, red color shows donor (IR55419–04) allele, blue color shows the introgressed DTY QTL regions, green color shows the SUB1 region, and dark gray lines show heterozygotes. Selection focus on presence of QTLs and overall phenotypic performance led to relatively lower background clarity but allowed selection of best performing NILs. (PDF 78.7 kb) [file 11032_2017_737_MOESM6_ESM.pdf]

A

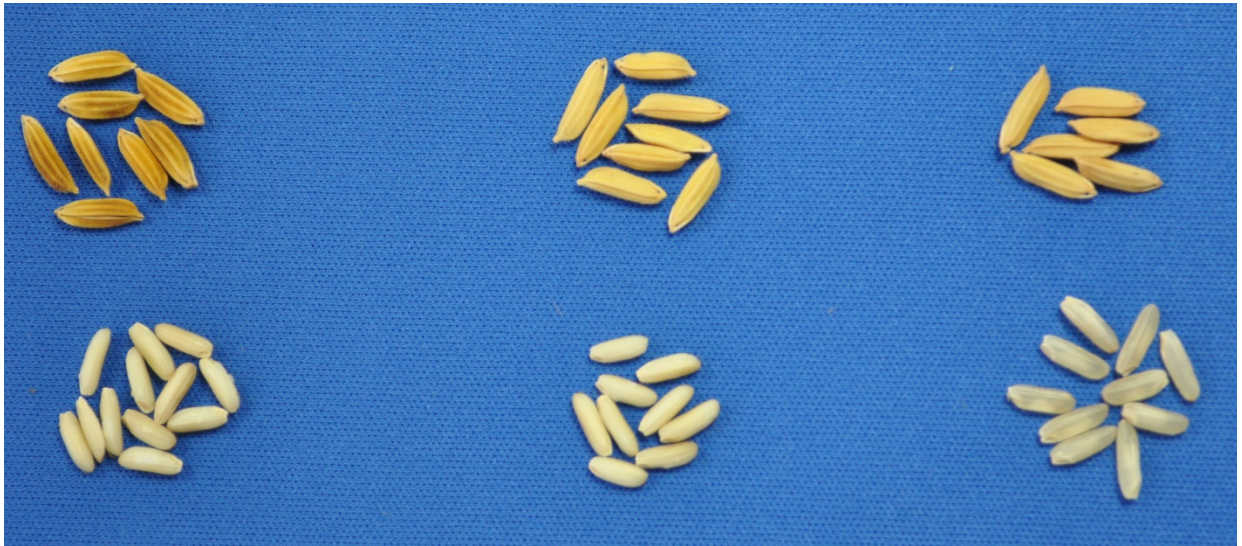

TDK1

TDK1-Sub1

IR102776-31-66-2-2-2

B

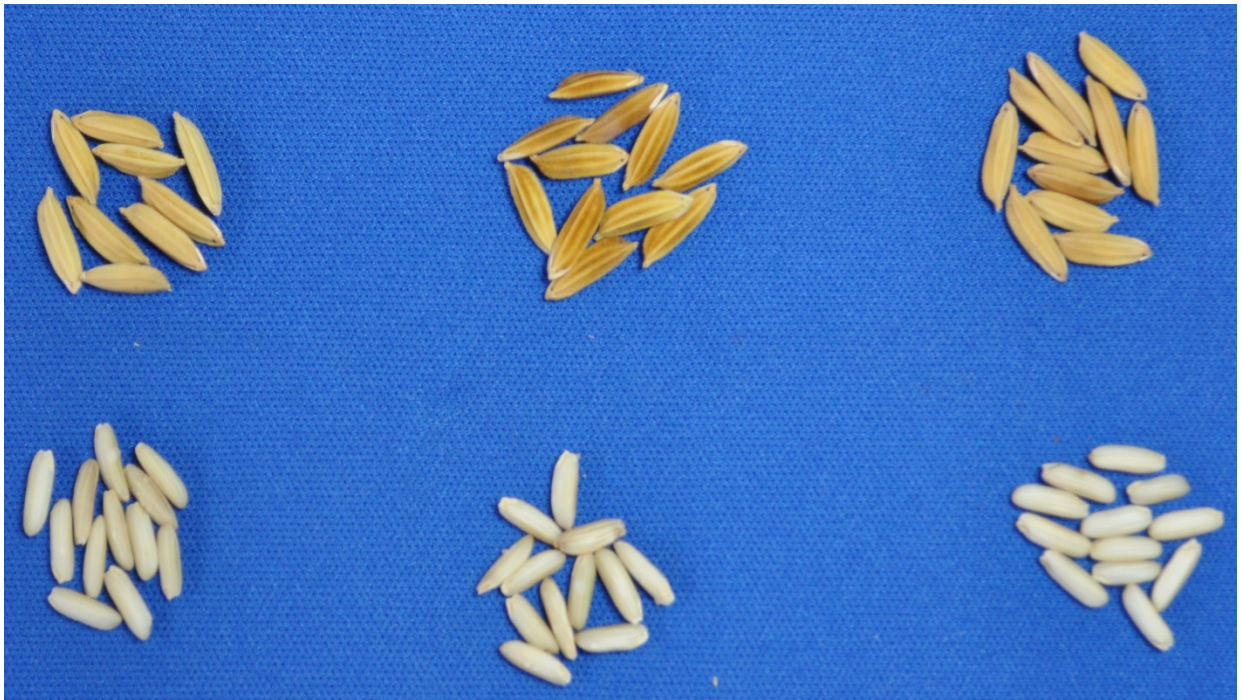

TDK1-Sub1

TDK1

IR102777-18-128-2-1-4

Supplement: Supplementary file 7 — Grain type variations obtained within the NILs compared to IR55419–04 (non- waxy) and TDK1/ TDK1 Sub1 (waxy). Waxiness ranged from intermediate to high in the NILs. Line IR102776–31–66-2-2-2 (a) showed intermediate waxiness while IR102777–18–128-2-1-4 had waxy grain type similar to TDK1 and TDK1 Sub 1. (PDF 1859 kb) [file 11032_2017_737_MOESM7_ESM.pdf]
