# Supplementary material for: Combining drought and submergence tolerance in rice: marker-assisted breeding and QTL combination effects
Source: Mol Breed. 2017 Nov 4;37(12):143. doi: 10.1007/s11032-017-0737-2 (PMC5670188; doi:10.1007/s11032-017-0737-2)
Supplement: Supplementary file 8 — SSR genotyping protocol used for foreground selection during the NIL development process. (DOCX 19.5 kb) [file 11032_2017_737_MOESM8_ESM.docx]

1. **SSR genotyping protocol used for foreground selection during the NIL development process**

For SSR genotyping, DNA was extracted from leaf samples collected from 2-week-old plants. Young-leaf samples were collected and freeze-dried. A Geno-grinder was used to grind these leaf samples and the DNA was extracted using a modified CTAB method **(Murray and Thomson, 1980)** in deep-well plates. A 0.8% agarose gel was used to check the quality and quantity of the extracted DNA and the samples were then diluted to a final concentration of 20 ng μL^−1^ with TE (Tris-EDTA) buffer. The method described by **Panaud et al. (1996)** was used to perform polymerase chain reaction (PCR). Upon completion of PCR, 4 μL of 6X loading dye was added to the product and 4 μL of this solution mix was loaded into an 8% (w/v) polyacrylamide gel **(Sambrook et al., 1989)** for size separation of the amplified DNA fragments. The mini vertical electrophoresis system (CBS Scientific, model MGV-202–33) was used to conduct the electrophoresis. SYBR® Safe gel stain (Invitrogen) was used to stain the separated DNA fragments. The fragments were then visualized using a UV trans-illuminator.

Murray, M.G., Thompson, W.F., 1980. Rapid isolation of high molecular weight plant DNA. Nucl. Acids Res. 8, 4321–4326.

Panaud, O., Chen, X., McCouch, S., 1996. Development of microsatellite markers and characterization of simple sequence length polymorphism (SSLP) in rice (*Oryza sativa* L.). Mol. Gen. Genet. 252, 597–607.

Sambrook, J., Fritsch, E.F., Maniatis, T., 1989. Molecular cloning: a laboratory manual. 2nd edn. Cold Spring Harbor, New York, USA.
